# New specimens of the early Permian apex predator *Varanops brevirostris* at Richards Spur, Oklahoma, with histological information about its growth pattern

**DOI:** 10.7717/peerj.14898

**Published:** 2023-02-15

**Authors:** Tea Maho, Joseph J. Bevitt, Robert R. Reisz

**Affiliations:** 1 University of Toronto, Mississauga, Ontario, Canada; 2 International Centre of Future Science, Dinosaur Evolution Research Center, Jilin University, Changchun, China; 3 Australian Centre for Neutron Scattering, Australian Nuclear Science and Technology Organisation, Lucas Heights, New South Wales, Australia

**Keywords:** Histology, Permian, Computed tomography, Varanopid, Postcranial anatomy, Age determination

## Abstract

An articulated pelvic region and additional isolated material of *Varanops brevirostris*, which are indistinguishable from those of the generotype from the *Cacops* bonebed, demonstrate the presence of this large varanopid at the Richards Spur locality. The articulated specimen includes lumbar, sacral, and anterior caudal vertebrae, partial pelvis, femur, and proximal part of tibia, confirming the autapomorphies previously suggested for this species. These include the presence of distinct blade-like shapes of the neural spines in the sacral region, the presence of deeply excavated pubis, and the presence of a distinct transverse ridge on the ventral surface of the femur distal to the intertrochanteric fossa. It has also been found that the transverse ridges and grooves become larger during ontogeny since the juvenile specimen did not exhibit a well-developed ridge. Histological analysis of isolated limb bones and neutron computed tomography (nCT) of the articulated specimen indicate that the latter likely belonged to an adult individual. This is in contrast to the other varanopid at Richards Spur, the significantly smaller, more gracile predator *Mesenosaurus efremovi*, which also shows the presence of growth lines and the external fundamental system with an estimated minimum age of fourteen.

## Introduction

*Varanops*, a member of the clade Varanopidae, is a large early Permian carnivorous synapsid reaching up to 1.5 m in length. Initially identified and described as *Varanosaurus brevirostris* from the *Cacops* bonebed, near Indian Creek, Baylor County in Texas (Williston, 1910, 1911), it was later recognized as a distinct new genus, *Varanops brevirostris* (Williston, 1914; Watson, 1914). Few specimens of this taxon have been thoroughly described to date, with the most recent being the cranial and postcranial material discovered in the Mud Hill locality, Texas (Campione & Reisz, 2010).

The Dolese Brothers limestone quarry near Richards Spur, Oklahoma, preserves abundant fossil material from the early Permian, a pivotal time in the early evolution of terrestrial tetrapods (MacDougall et al., 2017). This is a taxonomically rich fossil assemblage, one of the richest and most diverse for the Paleozoic, with the vast majority of the faunal composition being small terrestrial vertebrates, exemplified by the small *Doleserpeton* (Gee, Haridy & Reisz, 2020; Sigurdsen & Bolt, 2010) and other small non-amniotic vertebrates, small captorhinid and parareptile species of amniotes (deBraga, Bevitt & Reisz, 2019; Kissel, Dilkes & Reisz, 2002), and a relatively small unnamed sphenacodontid (Evans, Maddin & Reisz, 2009). Two small to medium-sized varanopids, *Mesenosaurus* (Maho, Gee & Reisz, 2019) and *Mycterosaurus* (Reisz, Wilson & Scott, 1997), have also been identified. Within the locality, larger individuals have also been found, but mostly the remains of the dissorophids *Acheloma* (Sullivan, Reisz & May, 2000; Polley & Reisz, 2011) and *Cacops* (Fröbisch & Reisz, 2012; Reisz, Schoch & Anderson, 2009). Other larger taxa are represented by very rare, isolated, fragmentary skeletal elements and teeth of the synapsid *Dimetrodon* (Brink, MacDougall & Reisz, 2019), and what appears to be a large varanopid similar to *Varanops* (Maddin, Evans & Reisz, 2006).

In contrast to more typical floodplain deposits that characterize most of the early Permian fossiliferous localities, Richards Spur represents an upland natural trap that captured vertebrate remains in fissure fills that formed an extensive cave system (MacDougall et al., 2017). This has resulted not only in high-quality preservation but also unusually large sample sizes for many taxa from the early Permian. In addition, varanopids have been generally known to be monospecific for individual localities, with each early Permian locality yielding one species. In contrast, Richards Spur has yielded multiple species of varanopids (Huttenlocker & Shelton, 2020; MacDougall et al., 2017), including *Mycterosaurus* represented by a fragmentary jaw (Reisz, Wilson & Scott, 1997), *Mesenosaurus* known from several skulls and partial skeletons (Maho, Gee & Reisz, 2019), and limited fragmented material of a large varanopid that may have been *Varanops* (Maddin, Evans & Reisz, 2006).

Recently uncovered new specimens from the Dolese Brothers Limestone Quarry near Richards Spur, including for the first-time articulated materials, can be assigned with confidence to *Varanops* and are described here, demonstrating unequivocally the presence of this taxon at Richards Spur. We used neutron computed tomography (nCT) to describe the new well-preserved postcranial material that could not be exposed through normal preparation. Additional postcranial material, including vertebrae and humeri, have been prepared and scientifically illustrated. Partial femoral material of this taxon has also been sampled histologically, allowing us to evaluate the growth pattern of this gracile predator. Moreover, the excellent preservation of the material allows us to compare the new material to the previously described specimens from the *Cacops* bonebed.

## Materials and Methods

### Neutron Computed Tomography (nCT) Images

Similar to Mooney et al. (2022), neutron tomographic data was collected for ROMVP 87380 using the DINGO thermal-neutron imaging instrument (Garbe et al., 2015), which is located and tangentially facing the 20 MW Open-Pool Australian Lightwater (OPAL) reactor, housed at the Australian Nuclear Science and Technology Organisation (ANSTO), Lucas Heights, New South Wales, Australia.

For this study, a high-flux mode was configured for DINGO, with a collimation ratio (*L*/*D*) of 1,000, with *L* being neutron aperture-to-sample length and *D* being the neutron aperture diameter, which yielded an approximate flux at sample of 1.15 × 10^7^n∙cm^−2^s^−1^ (Garbe et al., 2015). A Teledyne Photometrics Iris 15™ large field of view scientific cMOS camera (16-bit, 5,056 × 2,960 pixels) was used with a Makro Planar 24 mm Carl Zeiss lens and a 100 μm thick scintillator screen (ZnS/^6^LiF, RC Tritec AG), which produced a 73.3 × 73.3 μm pixel size and 200 × 200 mm^2^ field of view.

A total of 720 equally-spaced angle shadow-radiographs which were obtained every 0.25° as the sample was rotated 180° (about its vertical axis). The specimen’s rotation axis was positioned 80 mm from the detector’s face. Prior to shadow-radiograph acquisition, both dark (closed shutter) and beam profile (open shutter) images were obtained for calibration. To reduce anomalous noise, a total of three individual radiographs with an exposure length of 8 s were acquired at each angle (Mays, Bevitt & Stilwell, 2017), resulting 5.7 h for the total scan time.

Using ImageJ v.1.51h after scan completion, the individual radiographs were summed in post-acquisition processing using the ‘Grouped Z Project’ function while also removing anomalous white spots using the threshold filter. Normalisation and tomographic reconstruction of the 16-bit raw data was performed using Octopus Reconstruction v.8.8 (Inside Matters NV), resulting in virtual slices perpendicular to the rotation axis (Mooney et al., 2022).

Using a Canberra Radiagem Survey Meter Dosimeter dose, the neutron radioactivation of the specimen was recorded 30 min post-scan, yielding a 30 s average dose rate on contact of 85 mSv/h. Lastly, after 7 days, no residual radioactivity was recorded, allowing the specimen to be cleared for return.

Using ImageJ (version 1.53a), all of the 16-bit TIFF slices from the scanned *Varanops* block were converted to 8-bit. These slices were then stacked in an image sequence and later rendered and segmented using Avizo Lite (version 2020.3) registered to R.R. Reisz at the University of Toronto Mississauga.

### Histology

Histological analysis was completed following Padian & Lamm (2013). Three *Varanops* (ROMVP 87396, ROMVP 87797, ROMVP 87798) and one *Mesenosaurus* (ROMVP 87799) proximal femora were first photographed and then embedded using Castolite AP polyester resin, later vacuumed, and left to cure for approximately 24 h. Transverse cross-sections were performed on the femora, either near the proximal end (below the intertrochanteric fossa) or the midshaft, using the Metcut-5 low-speed saw (MetLab) with a diamond wafer blade (225 rpm). After the initial cut, the specimens were glued on frosted plexiglass slides and cut a second time. Later, the specimens were ground using first the Metcut-10 Geo (MetLab) machine with a grinding cup and second progressively finer grit (1000- to 2000-grit) silicon carbide paper. Using a Nikon DS-Fi1 camera mounted onto a Nikon AZ-100 microscope with the NIS Elements-Basic Research software (registered to R.R. Reisz of the University of Toronto Mississauga) the specimens were photographed. The photographs were then analyzed to identify cyclic growth marks, including lines of arrested growth (LAGs) and annuli, defined as a slowdown of bone deposition shown by faint zones of denser bone within the cortex of the femora (Huttenlocker, Woodward & Hall, 2013). The terminology for the growth marks, tissue types, and vascular canals is consistent with Francillon-Vieillot et al. (1990), Huttenlocker, Woodward & Hall (2013), McFarlin (2006), and McFarlin et al. (2016). Table 1 provides a list of the long bone material with measurements.

**Table 1:** *Varanops* and *Mesenosaurus* long bone specimen and thin section numbers (TSN), including measurements and growth marks (LAGs and annuli) for each element.

Taxon name	Specimen number	TSN	Element	Length (mm)	Proximal width (mm)	Shaft width (mm)	Distal width (mm)	Growth marks
*Varanops brevirostris*	ROMVP 87380	None	Femur	100.2	26.7	10.2	28.9	NA
*Varanops brevirostris*	ROMVP 87396	TS01772	Femur	NA	26.6	12.6	NA	12
*Varanops brevirostris*	ROMVP 87797	TS01765	Femur	NA	25.9	11.8	NA	10
*Varanops brevirostris*	ROMVP 87798	TS01766	Femur	NA	25.5	10.1	NA	11
*Varanops brevirostris*	ROMVP 87802	None	Femur	NA	22.5	NA	NA	NA
*Mesenosaurus* sp.	ROMVP 87799	TS01768	Femur	NA	17.2	7.2	NA	13
*Varanops brevirostris*	ROMVP 87397	None	Humerus	69.4	31.6	7.7	35.4	NA
*Varanops brevirostris*	ROMVP 87398	None	Humerus	NA	NA	NA	42	NA
*Varanops brevirostris*	ROMVP 87380	None	Tibia	NA	21.3	7.7	NA	NA

## Systematic paleontology

SYNAPSIDA Osborn, 1903

EUPELYCOSAURIA Kemp, 1982

VARANOPIDAE Romer & Price, 1940

VARANODONTINAE Reisz and Berman, 2001

*VARANOPS* Williston, 1914

**Type Species**—*Varanops brevirostris* (Williston, 1911).

**Diagnosis**—As for type species.

**Holotype**—FMNH (WM) 644, from the *Cacops* bonebed, Indian Creek, west of Coffee Creek, Baylor County, Lower Permian.

**Horizon and Locality**—Dolese Brothers Limestone Quarry near Richards Spur, Oklahoma, U.S.A. Early Permian.

**Referred Specimens**— ROMVP 87380, postcranial material including complete femur, partial tibia, partial pelvic girdle, partial vertebral column; OMNH 73502, dorsal vertebrae; ROMVP 87397, complete humerus; ROMVP 87398, partial humerus; ROMVP 87396, partial proximal end of femur; ROMVP 87797, partial proximal end of femur; ROMVP 87798, partial proximal end of femur; ROMVP87802, partial proximal end of femur; ROMVP 87799, partial proximal end of femur of *Mesenosaurus*.

## Results

### Axial skeleton

**Figure 1:**
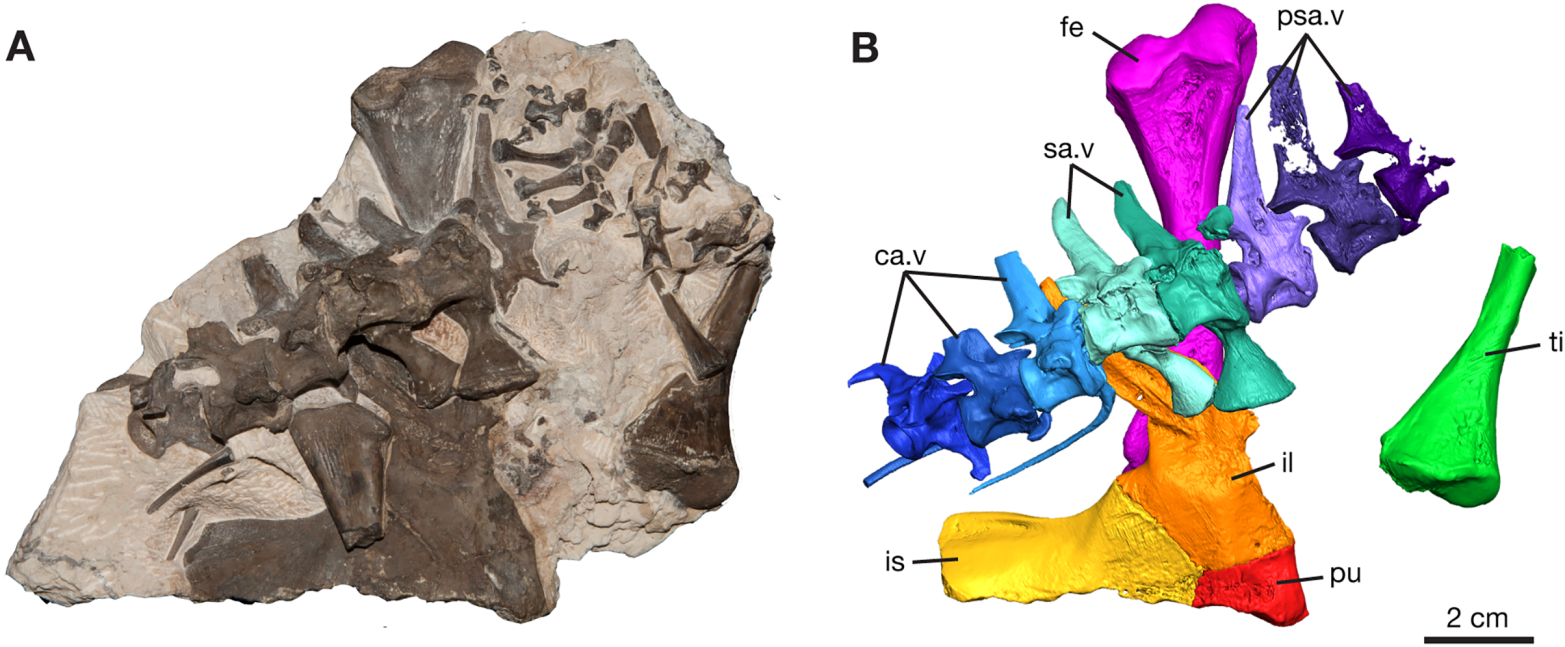
*Varanops* block with postcrania, ROMVP 87380. (A) Photograph and (B) digital rendering. Abbreviations: ca.v, caudal vertebrae; fe, femur; il, ilium; is, ischium; pu, pubis; psa.v, presacral vertebrae; sa.v, sacral vertebrae; ti, tibia.

**Figure 2:**
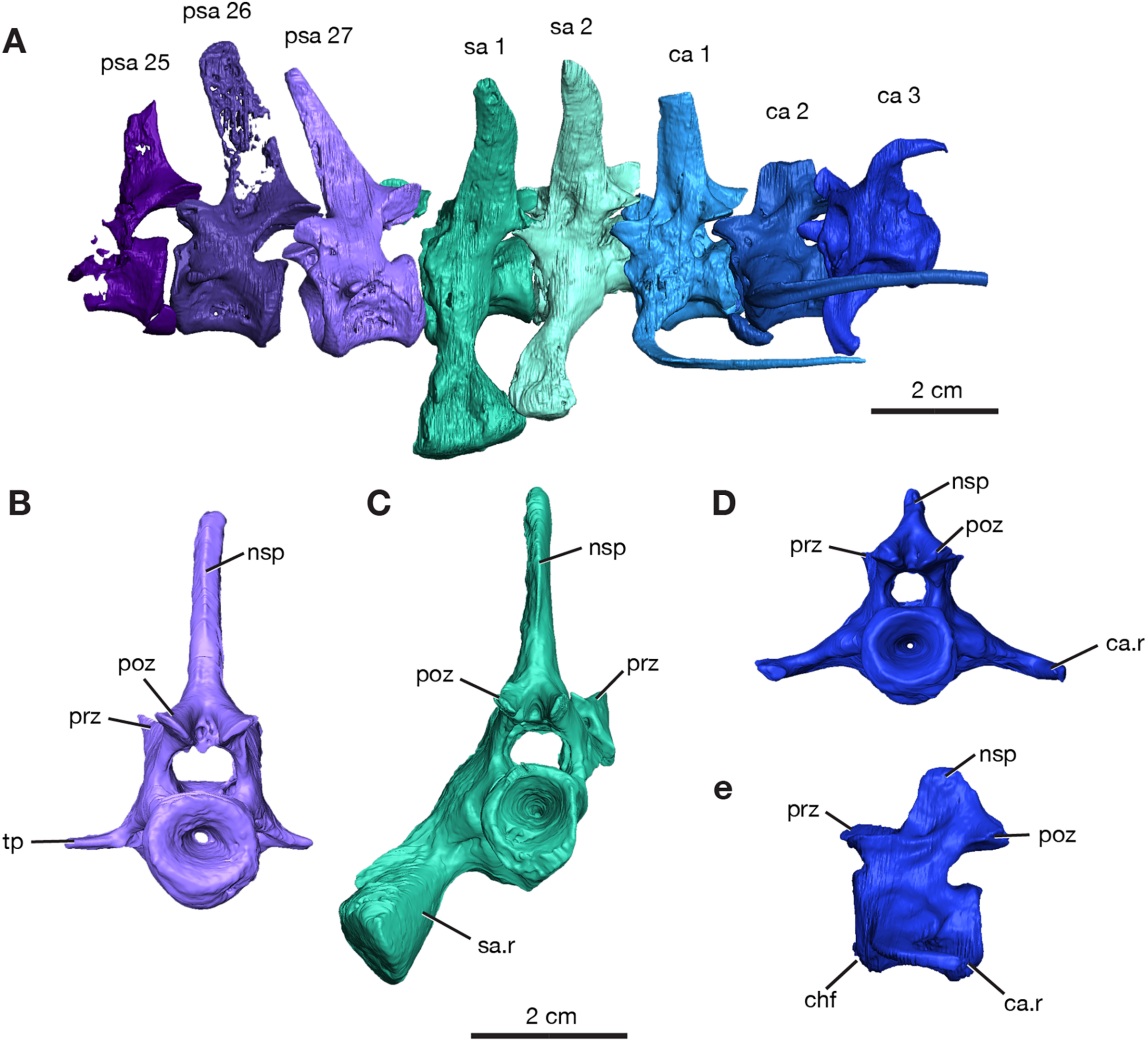
Rendering of *Varanops* ROMVP 87380 vertebrae. Posterior vertebrae including (A) last three presacral (25 to 27), two sacral, and the first three caudal. (B) Last presacral, psa27, in posterior view. (C) First sacral, sa1, in posterior view. (D) Third caudal, ca3, in posterior view and (E) right lateral view. Abbreviations: ca, caudal vertebrae; ca.r, caudal rib; chf, chevron facet; nsp, neural spine; poz, postzygapophysis; prz, prezygopophysis; psa, presacral; sa, sacral vertebrae; sa.r, sacral rib; tp, transverse process.

The most significant discovery is represented by ROMVP 87380, where eight vertebrae of the vertebral column are composed of the last three presacral vertebrae, two sacral vertebrae, and three anterior-most caudal vertebrae (Figs. 1 and 2). Although most of the presacral and caudal vertebral column is missing, since Williston (1911) noted a total of twenty-seven presacral, two sacral, and forty-seven caudal vertebrae for the holotype of *Varanops brevirostris*, the preserved part of the column is in near perfect articulation.

**Figure 3:**
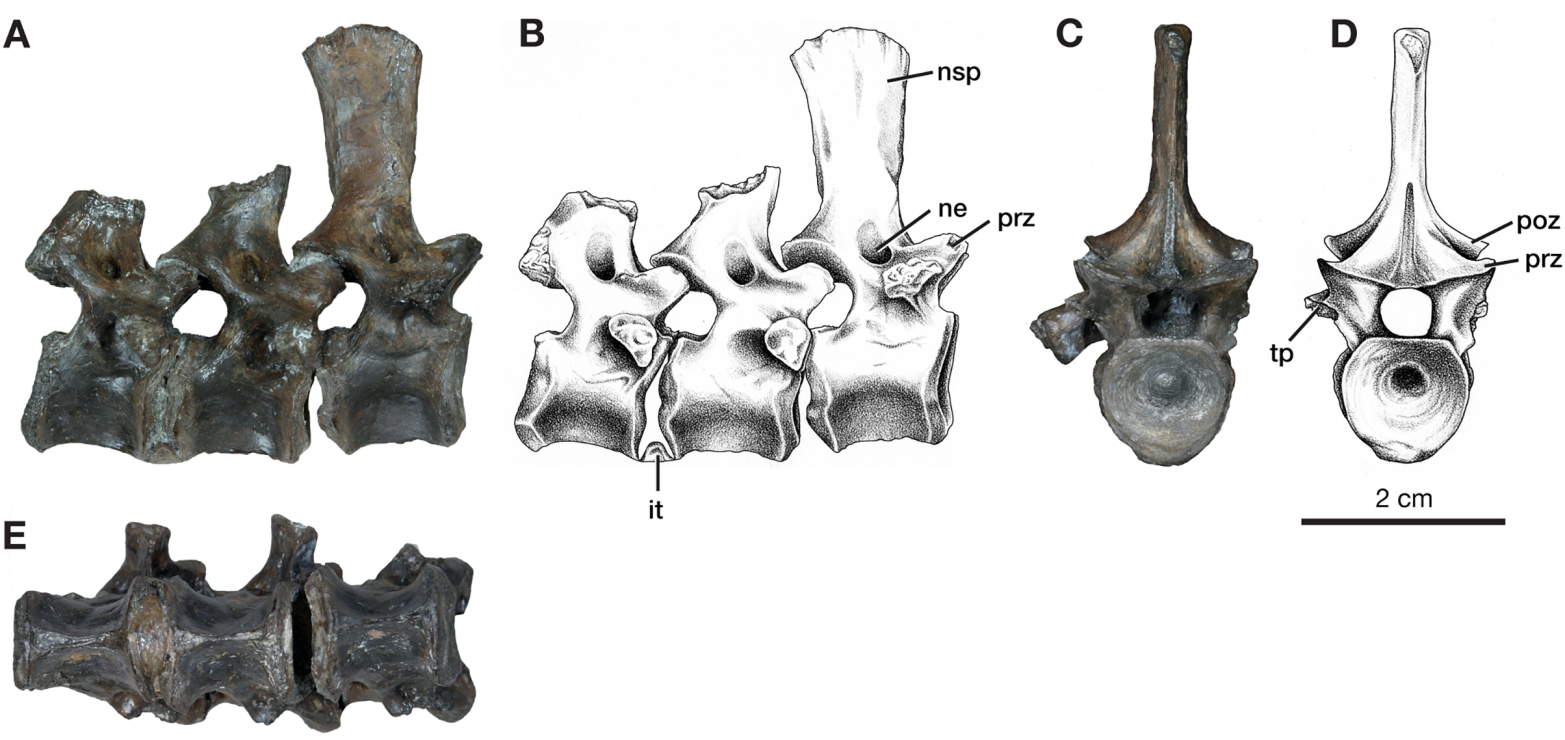
Dorsal vertebrae of *Varanops* OMNH 73502. (A) Photograph and (B) illustration of lateral view. (C) Photograph and (D) illustration of anterior view. (E) Photograph of ventral view. Abbreviations: it, intercentrum; ne, neural spine excavation; nsp, neural spine; poz, postzygapophysis; prz, prezygopophysis; tp, transverse process.

The neural spines for the last three presacrals are positioned slightly forward and the twenty-seventh presacral has the only complete neural spine, illustrating the dorsal tapering with a smaller anteroposterior width at the top (Fig. 2A), compared to the anterior presacral neural spines, which are vertical (Fig. 3). This forward position has also been noted by Williston (1911) and Campione & Reisz (2010). The posterior dorsal vertebrae have a less excavated base of the neural spine when compared to the sacral vertebrae. The last three presacral vertebrae could be considered to be true lumbars since no ribs appear to be attached to the very delicate, slender transverse processes. Their shape and size suggest that there were no ribs in this region of the column. Thin transverse processes are present on the last three presacral vertebrae, with a slight decrease in length occurring posteriorly. The V-shape space in ROMVP 87380 (Fig. 2) created between the posterior edge of the last presacral and the anterior edge of the first sacral neural spine is a diagnostic feature for *Varanops brevirostris* and is clearly well preserved in the Richards Spur specimen. The centra of the lumbar vertebrae are uniformly shaped and the keels are more rounded.

Two sacral vertebrae are identifiable from the short and massive ribs preserved on the left lateral side. The rib on the first sacral is the largest and extends posteriorly, while the second sacral rib extends anteriorly and is attached to the first (Fig. 2A). This morphology has similarly been noted for the holotype (Williston, 1911). The neural spine of the first sacral is positioned posteriorly and tapers dorsally. There are deep, elongated excavations on the base of the neural spines. For the sacral vertebrae, the prezygapophyses are widely separated, while the postzygapophyses are narrower and in line with the caudal vertebrae. Interestingly, the first sacral vertebra has a rounded ventral ridge that is actually more slender than the massive rounded ventral ridge of the last presacral vertebra; the second sacral vertebra has an even more slender ventral ridge compared to the first sacral. The first three caudal vertebrae have centra that are more cylindrical, with a broader and more rounded keel compared to the dorsal vertebrae. From the first to the third caudal vertebra, the keel transitions to becoming sharper. Only the first caudal vertebra preserves a complete neural spine, has a smaller height than the sacral spines, and it is oriented vertically. Similar to the posterior dorsal vertebrae, excavation of the neural spines of the caudal vertebrae is present but more modest than those on the sacrals. No haemal (chevron) arches were preserved, but the facets are visible on the posteroventral surfaces of the centra (Fig. 3E). All three vertebrae have the ribs attached, with the first caudal (ca1; Fig. 2A) having a complete rib that extends far posteriorly and is oriented horizontally.

In addition to this specimen, three vertebrae found separately, OMNH 73502 (Fig. 3), can be interpreted as mid-dorsals, due to the shape and location of the transverse processes and the shape of the neural spines. These three vertebrae exhibit typical *Varanops* structures, with the best-preserved neural spine being flat and thin, having a rectangular outline in lateral view, with a broader anteroposterior top portion compared to the base (Fig. 3). The first vertebra in the series has a complete neural spine and broken transverse processes, whereas the most posterior vertebra has a broken neural spine and one complete transverse process with a teardrop shape at the articulating surface. The central articulating surfaces are greater in height anteriorly than posteriorly. The anterior vertebra has a sharp keel and a greater ventrolateral excavation resulting in a mid-ventral ridge, while the posterior vertebra has transitioned to a broader keel with a slightly flattened ventral surface (Fig. 3E). Deep neural excavations are present between the prezygapophyses and postzygapophyses of all three vertebrae.

### Forelimb

#### Humerus

**Figure 4:**
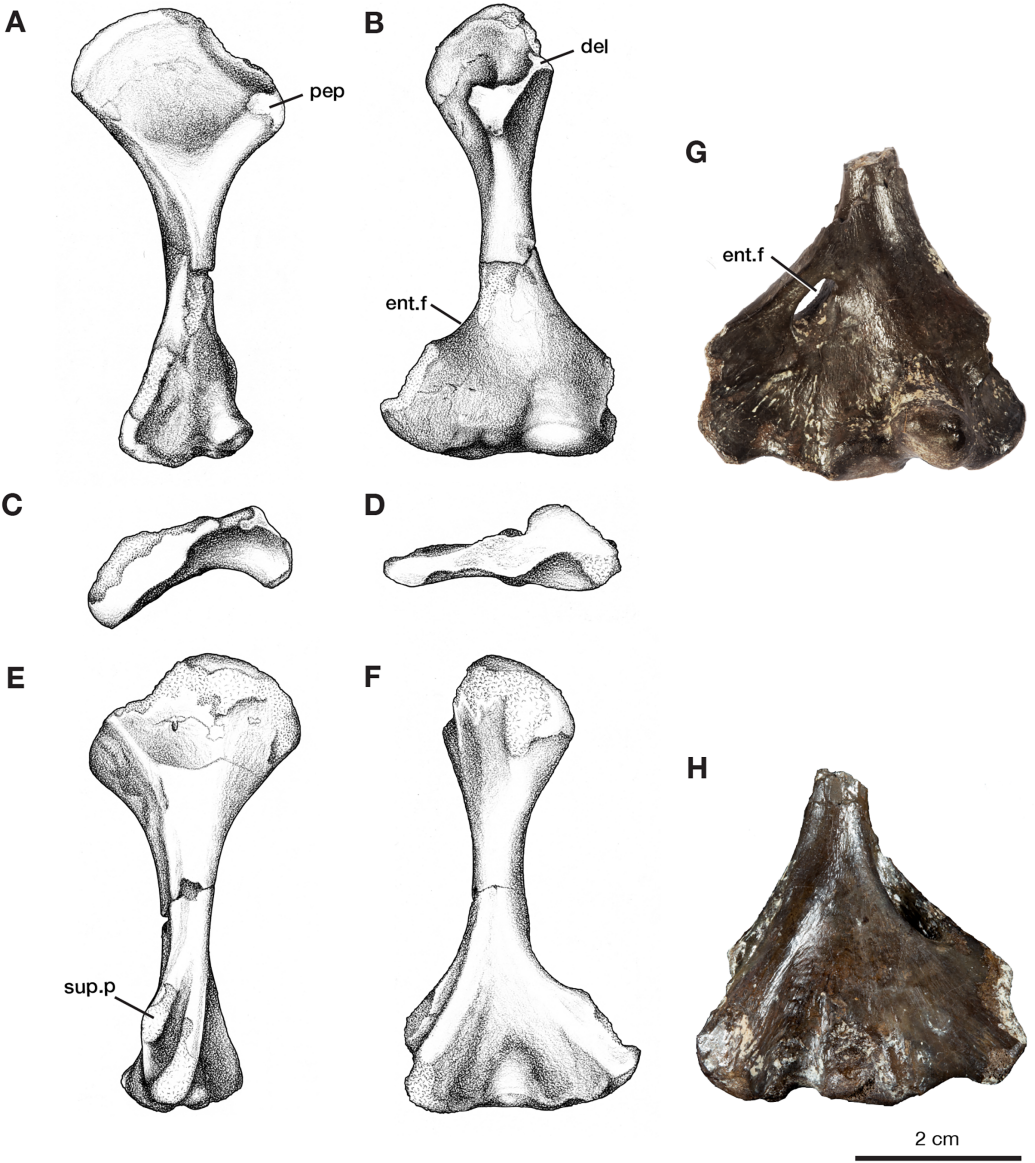
Left humeri of *Varanops*. Illustrations of complete humerus, ROMVP 87397, in (A) proximal dorsal, (B) distal dorsal, (C) proximal end, (D) distal end, (E) proximal ventral, and (F) distal ventral views. Photographs of partial humerus, ROMVP 87398, (G) distal dorsal and (H) distal ventral views. Abbreviations: del, deltoideus process; ent.f, entepicondylar foramen; pep, pectoralis process; sup.p, supinator process.

The left humerus, ROMVP 87397, has broadly expanded proximal and distal ends, with a thin, rounded shaft (Figs. 4A–4F). The length of the humerus is slightly smaller than the holotype described by Williston (1911); however, this may be a reflection of its juvenile status as indicated by the smaller and more poorly ossified articular surfaces, including those for the radius and ulna. Proximally the articular surface for the glenoid fossa is offset from the long axis of the bone and is widely separated from the delto-pectoral crest. The earlier ontogenetic state of ROMVP 87397 is apparent when compared to the much larger partial left humerus ROMVP 87398, which is composed of only the distal end and has well-ossified articular surfaces (Figs. 4G–4H). At the distal end of the humerus, the entepicondylar foramen is represented in ROMVP 87397 by its medial edge but the distal edge is not preserved (Fig. 4), while ROMVP 87398 has a complete well-developed, lateromedially elongate foramen. Both indicate that the entepicondylar foramen was large and elongate in this taxon (Fig. 4C). Anterodistally, the supinator process, although slightly damaged, indicates that it was modestly developed, separated dorsally from the ectepicondyle by a deep groove, and extended mainly distally, and only modestly anteriorly as in other *Varanops brevirostris* specimens.

### Pelvic girdle

**Figure 5:**
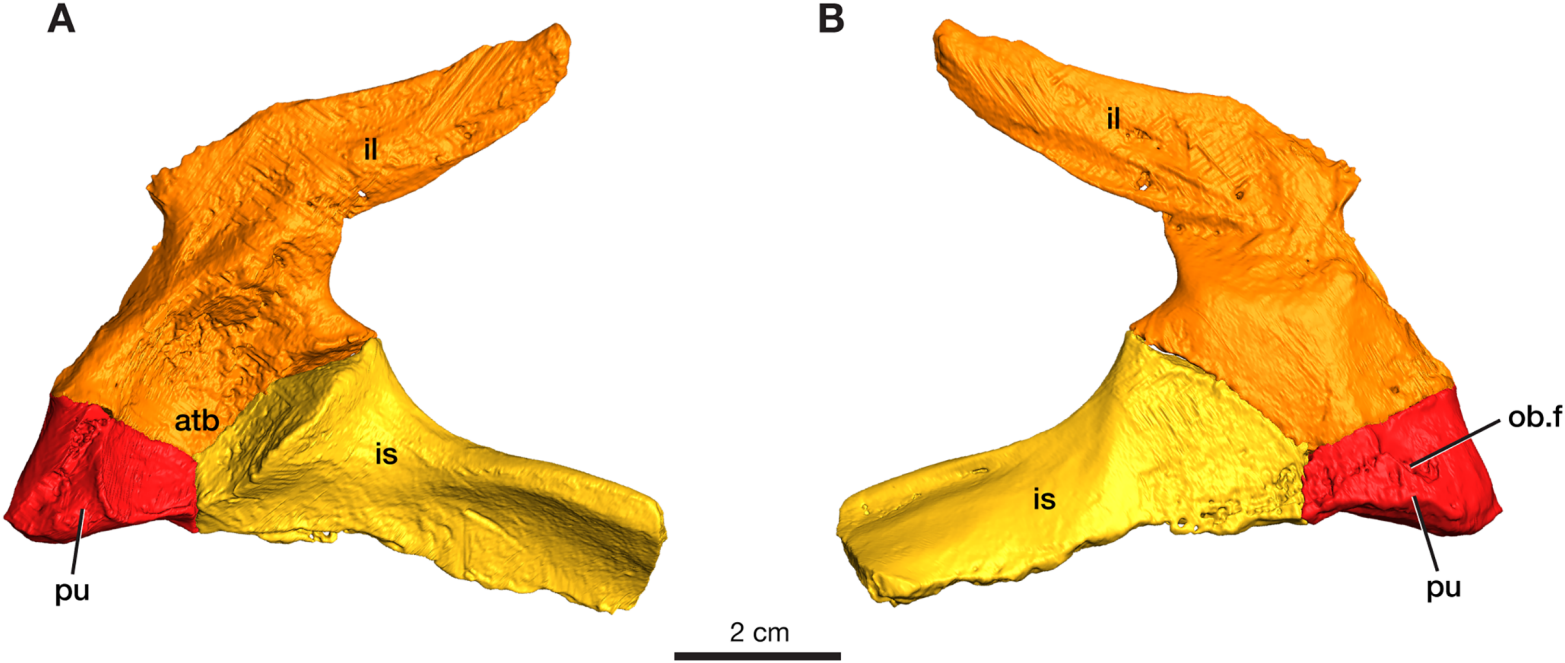
Rendering of *Varanops* ROMVP 87380 pelvic girdle. Left pelvis in (A) lateral and (B) medial view. Abbreviations: atb, acetabulum; il, ilium; is, ischium; pu, pubis; ob.f, obturator foramen.

The left pelvic girdle is preserved for ROMVP 87380 with the three elements (pubis, ilium, and ischium) in articulation with each other (Fig. 5).

### Pubis

The left pubis is incomplete in ROMVP 87380, with only the portion contributing to the acetabulum preserved; thus, the total length could not be determined. The pubic portion of the acetabulum appears to be oval in outline, and the start of the anterolateal ridge is visible. The obturator foramen is visible in medial view and extends laterally (Fig. 5). As in other varanopids, including *Varanops*, the pubis has an unusual twist relative to the broader pubo-ischiadic plate, being bent medially with a deep recess separating it from the anterolateral ridge. Interestingly, this morphology would create a much broader pubic space anteriorly than in other contemporary amniotes, where the pubo-ischiadic plate forms a single relatively flat continuous surface dorsally. However, the precise shape of the pubis is not readily discernible in this specimen, only the deep recess is preserved here, with the pubic portion of the ventral plate being largely lost. This morphology of the pubis is a distinctive feature of the derived varanopid pelves, being more pronounced in the varanodontines (Campione & Reisz, 2010), although also present in a more modest form in *Mesenosaurus* (RR Reisz, 2022, personal communication).

### Ilium

The left ilium is complete in ROMVP 87380, with the blade being long and narrow (Fig. 5). Similar to the condition in the holotype of *Varanops brevirostris* described by Williston (1911) and Maddin, Evans & Reisz (2006), the iliac blade tapers distally and the width of the neck and total length of the ilium is similar in size. The iliac blade projects backward and slopes gently dorsally, but no large extensions occur anteriorly. Instead, a small anterior process of the blade creates a concave anterior edge between the dorsal edge of the iliac blade and the body of the bone. The large acetabular surface faces mainly medially, with a slight ventrally facing upper region. The entire ventral border in the area of the acetabulum articulating to the pubis and ischium is similar to that seen in the holotype, while the overall acetabular component on the ilium has a rectangular outline where it meets the other pelvic elements. On the medial surface, well-developed facets for articulation with the sacral ribs are present with the larger one being for the first sacral rib. Similar to the holotype, a ridge that separates the second sacral rib from the axial musculature is present. The ventral edge of the iliac blade is slightly convex, as seen in the holotype, and curves upwards near its posterior end. The specimen is larger than the one previously ascribed to a juvenile *Varanops* (OMNH 73174; Maddin, Evans & Reisz, 2006) from this locality.

### Ischium

The left ischium is incomplete in ROMVP 87380 (Fig. 5), but the preserved morphology is similar to the holotype of *Varanops brevirostris* described by Williston (1911). The ischiadic portion of the acetabulum is significantly smaller than that of the ilium, it is short anteroposteriorly, but relatively tall and transversely massive, facing mainly anterolaterally. The bone is also fairly massive between its acetabular contribution and the posteriorly extending lateral ridge. The ventral surface of the ischiadic plate is concave medial and ventral to this ridge.

### Hind limb

#### Femur

**Figure 6:**
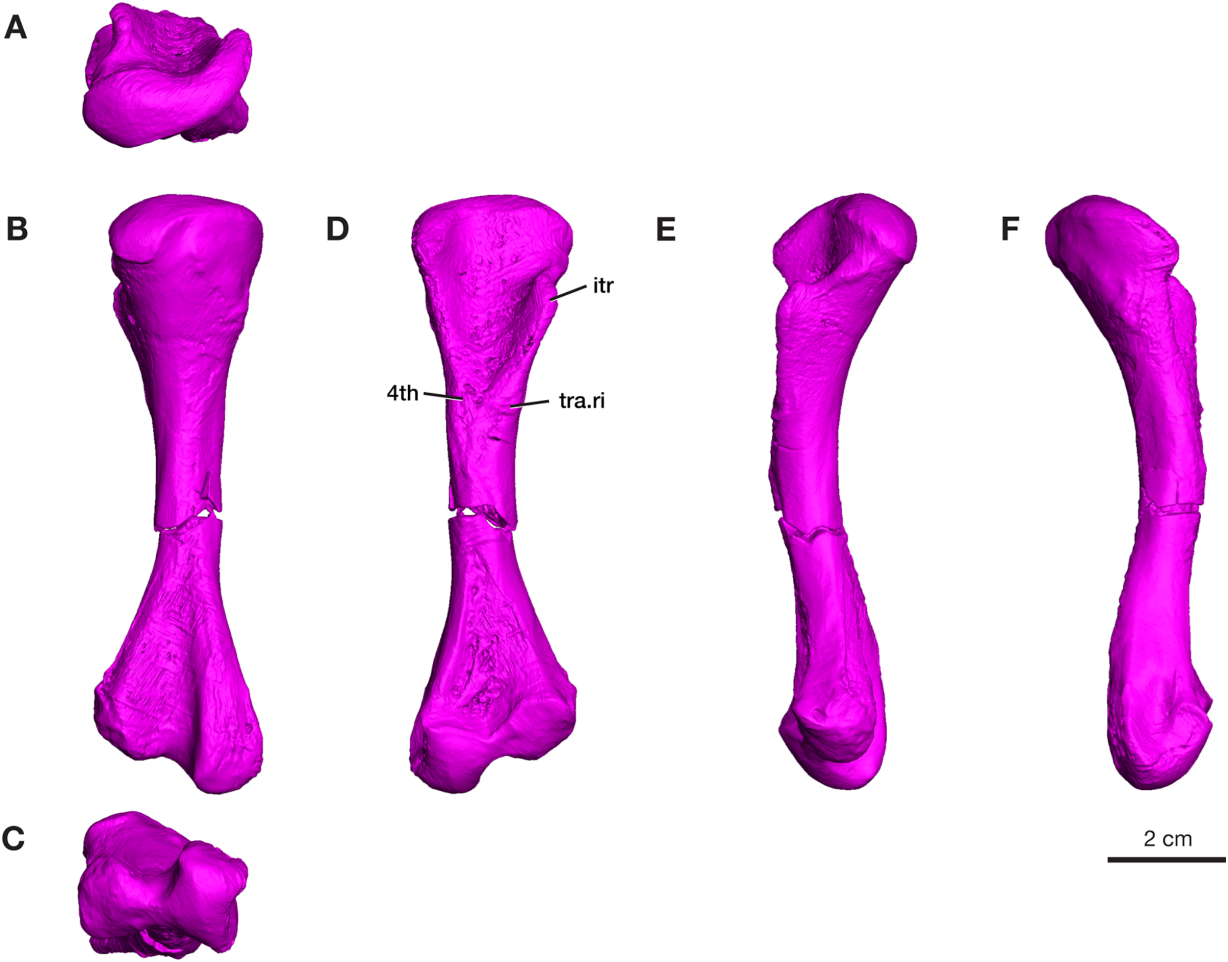
Rendering of *Varanops* ROMVP 87380 femur. Complete left femur showing (A) proximal, (B) dorsal, (C) distal, (D) ventral, (E) anterior, and (F) posterior views. Abbreviations: 4th, fourth trochanter; itr, internal trochanter; tra.ri, transverse ridge.

A complete left femur is preserved in ROMVP 87380, with a break in the middle of the shaft (Fig. 6). The total length of the femur is 100.2 mm, which is within the upper range of the specimens of *V. brevirostris* examined by Romer & Price (1940). On the proximal end, the intertrochanteric fossa is elongated and extends into the proximal region of the shaft, as previously described for the holotype (Romer & Price, 1940). The internal trochanter is located high and close to the proximal articulating surface and the ridge connecting it to the proximal articular surface is concave. Similar to the holotype (Williston, 1911), the shaft of the ROMVP 87380 femur is slender, 10.2 mm in diameter, and the fourth trochanter, usually extending distally from near the base of the intertrochanteric fossa is not prominent (Romer & Price, 1940; Reisz, 1986). Instead, the area of the 4^th^ trochanter carries a well-developed group of ridges and grooves extending transversely across the ventral surface of the bone. Although not found to be present in the *Cacops* bonebed materials, possibly caused by unfortunate preservational conditions combined with inferior preparation techniques employed in the early 20^th^ century studies (Williston, 1911; Romer & Price, 1940), this pattern of ridges and pockets are present in the well preserved *Varanops* femora from the Mud Hill locality in Texas (Campione & Reisz, 2010). The anterodorsal surface of the proximal articular surface extends farther distally than in the holotype. On the distal end, the posterior and anterior condyles are separated by a deep, elongated intercondylar fossa, and the anterior condyle is smaller than the posterior condyle. The posterior condyle has a deep groove along its posterior surface, which is a typical feature of varanopids. Both the proximal and distal ends are expanded and almost equal in width (26.7 and 28.9 mm), compared to the slender shaft region. The ends of the femur are about 28% of the total length. The midshaft is almost completely featureless, with a simple cylindrical shape and no distinguishing ridges. Additionally, the dorsal surface of the midshaft exhibits a slight concave curvature, similar to the holotype.

**Figure 7:**
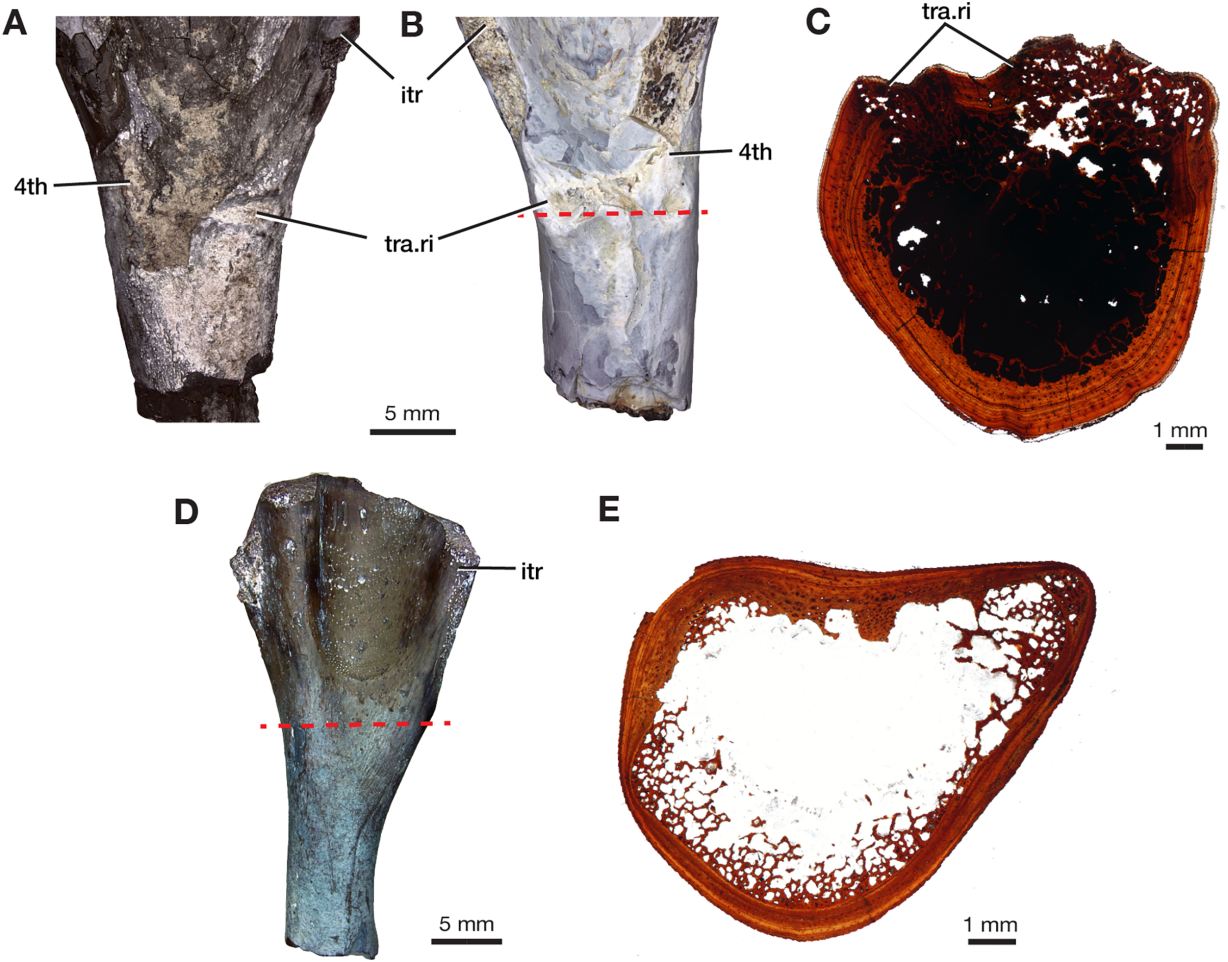
Morphology of lower intertrochanteric to midshaft region of femora. Photograph of (A) ROMVP 87797 and (B) ROMVP 87798 in ventral view, showing external surface of transverse ridge and red dotted line showing cut. (C) Cross-section of transverse ridge region. (D) Photograph of ROMVP 87799 ventral view, with red dotted line showing cut, and (E) Cross-section of lower intertrochanteric region. Abbreviations: 4th, fourth trochanter; itr, internal trochanter; tra.ri, transverse ridge.

The four additional proximal femora (ROMVP 87396, ROMVP 87797, ROMVP 87798, ROMVP87802) recovered from Dolese were found to have the distinctive transverse pocketing and ridges just distal the intertrochanteric fossa, with either one pit, or with the addition of the ridge near the lower portion of the internal trochanter (Figs. 7A–7B). This pocketing was also apparent through the nCT data of ROMVP 87380. ROMVP87802 appears to represent a more juvenile individual, smaller in size, with only one groove found distal to the intertrochanteric fossa.

### Tibia

**Figure 8:**
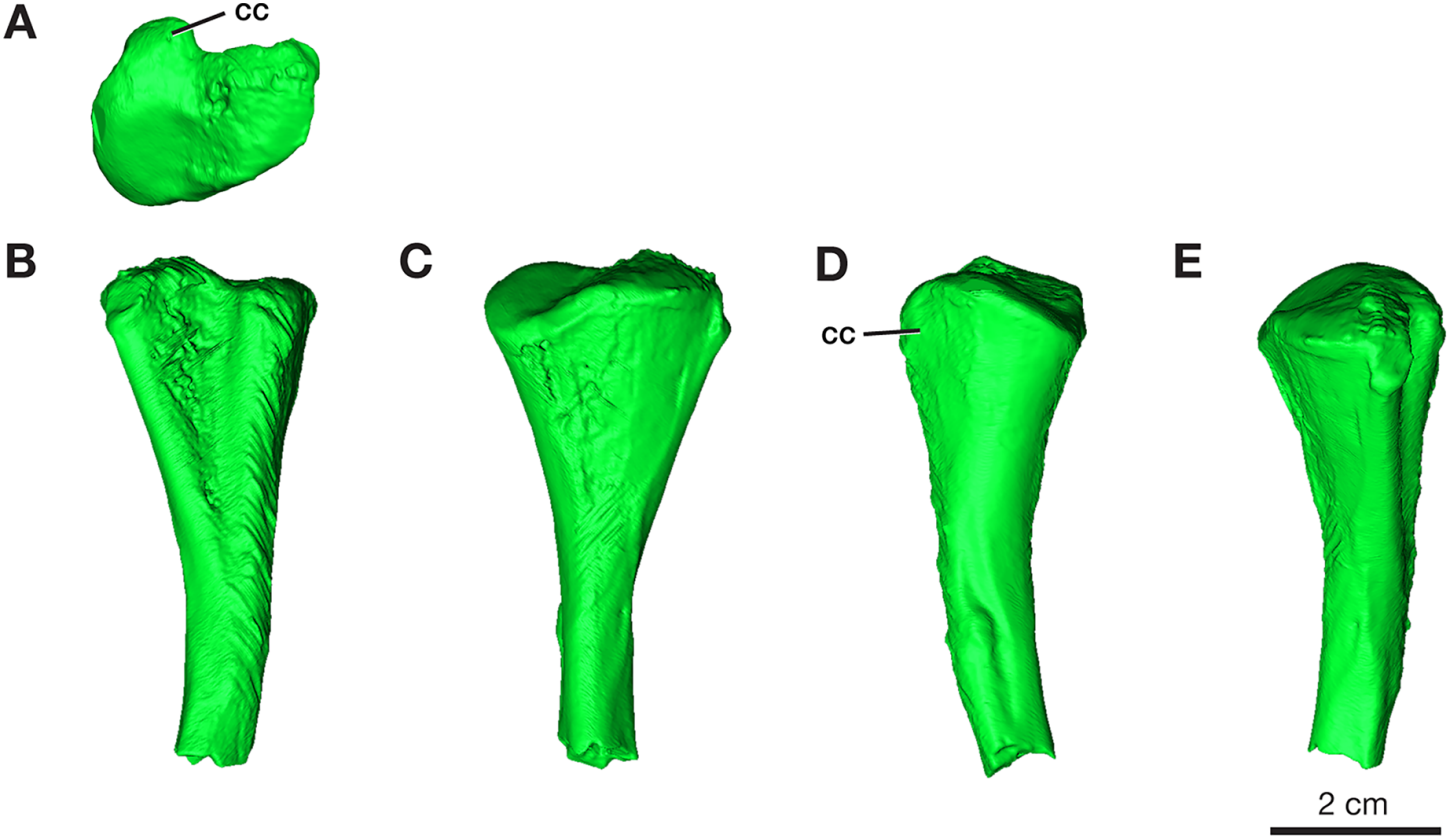
Rendering of *Varanops* ROMVP 87380 tibia. Proximal half of the right Tibia showing the (A) proximal, (B) lateral, (C) medial, (D) anterior, and (E) posterior views. Abbreviations: cc, cnemial crest.

Only the proximal half of the right tibia is preserved in ROMVP 87380, with a proximal width of 21.3 mm and a narrow shaft of 7.4 mm (Fig. 8). The shape and size of the preserved proximal region is similar to that seen in the holotype (Williston, 1911) and in the complete tibia previously reported from this locality (Maddin, Evans & Reisz, 2006). Although only half of the tibia is present, the slight medial bowing is visible in anterior and posterior views (Fig. 8), as seen in other *Varanops* specimens. The c-shaped outline of the articular surface observed in proximal view is formed from the well-developed cnemial crest on the lateral side. The total length was not directly determinable since the tibia is incomplete, but it appears to be slightly larger than the previously described tibia from this locality (OMNH 73175; Maddin, Evans & Reisz, 2006).

### Histological features of femora

**Figure 9:**
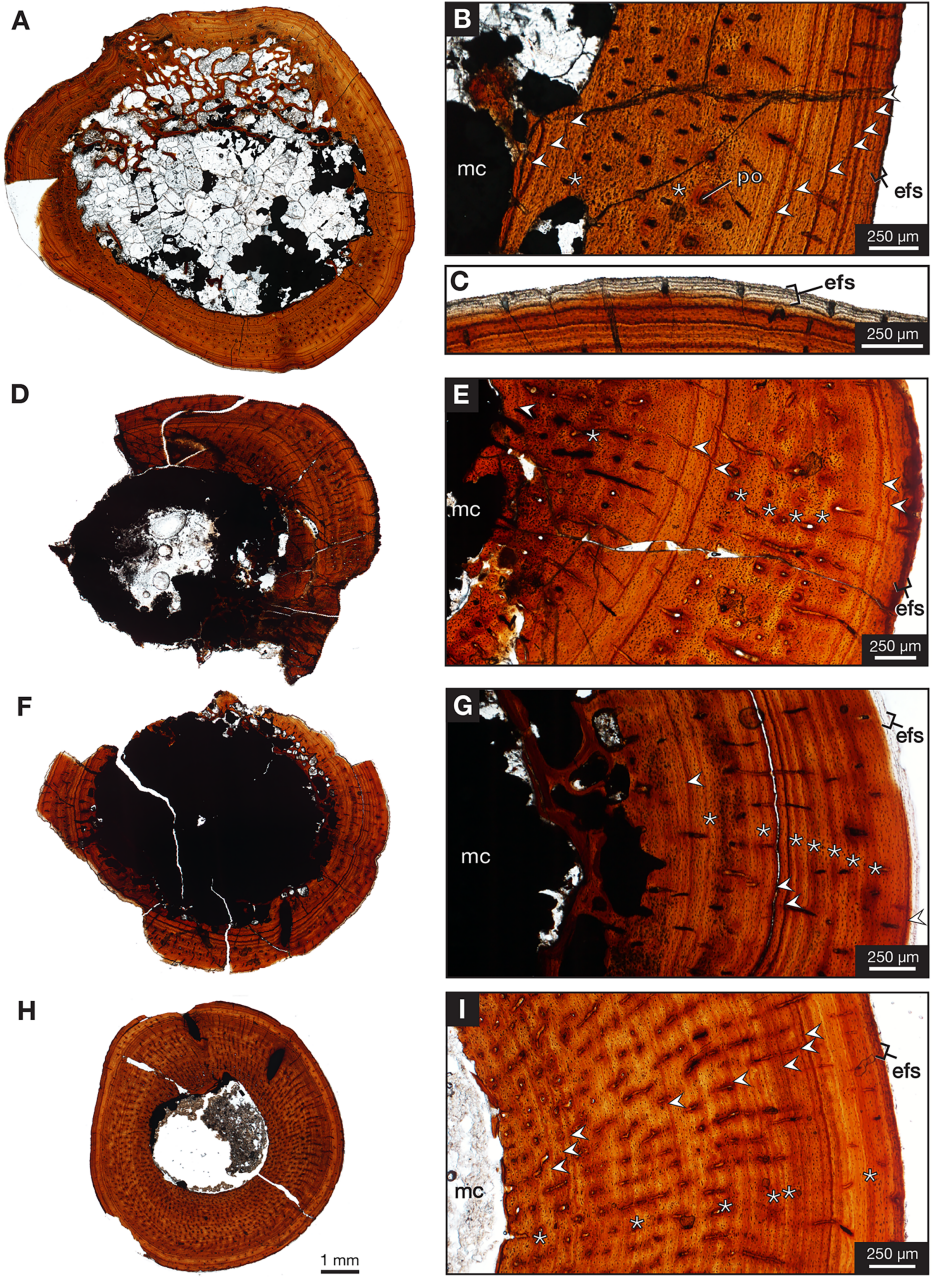
Midshaft histology of *Varanops* and *Mesenosaurus* femora. *Varanops* femur ROMVP 87396 (A) complete transverse cross-section, (B) close-up of midshaft showing growth lines, (C) close-up showing EFS. *Varanops* femur ROMVP 87797 (D) complete transverse cross-section, (E) close-up of midshaft showing growth lines. *Varanops* femur ROMVP 87798 (F) complete transverse cross-section, (G) close-up of midshaft showing growth lines. *Mesenosaurus* femur ROMVP 87799 (H) complete transverse cross-section, (I) close-up of midshaft showing growth lines. For the growth lines, white arrowheads represent lines of arrested growth (LAGs) and asterisks represent annuli. Abbreviations: efs, external fundamental system; mc, medullary (marrow) cavity; po, primary osteons.

**Figure 10:**
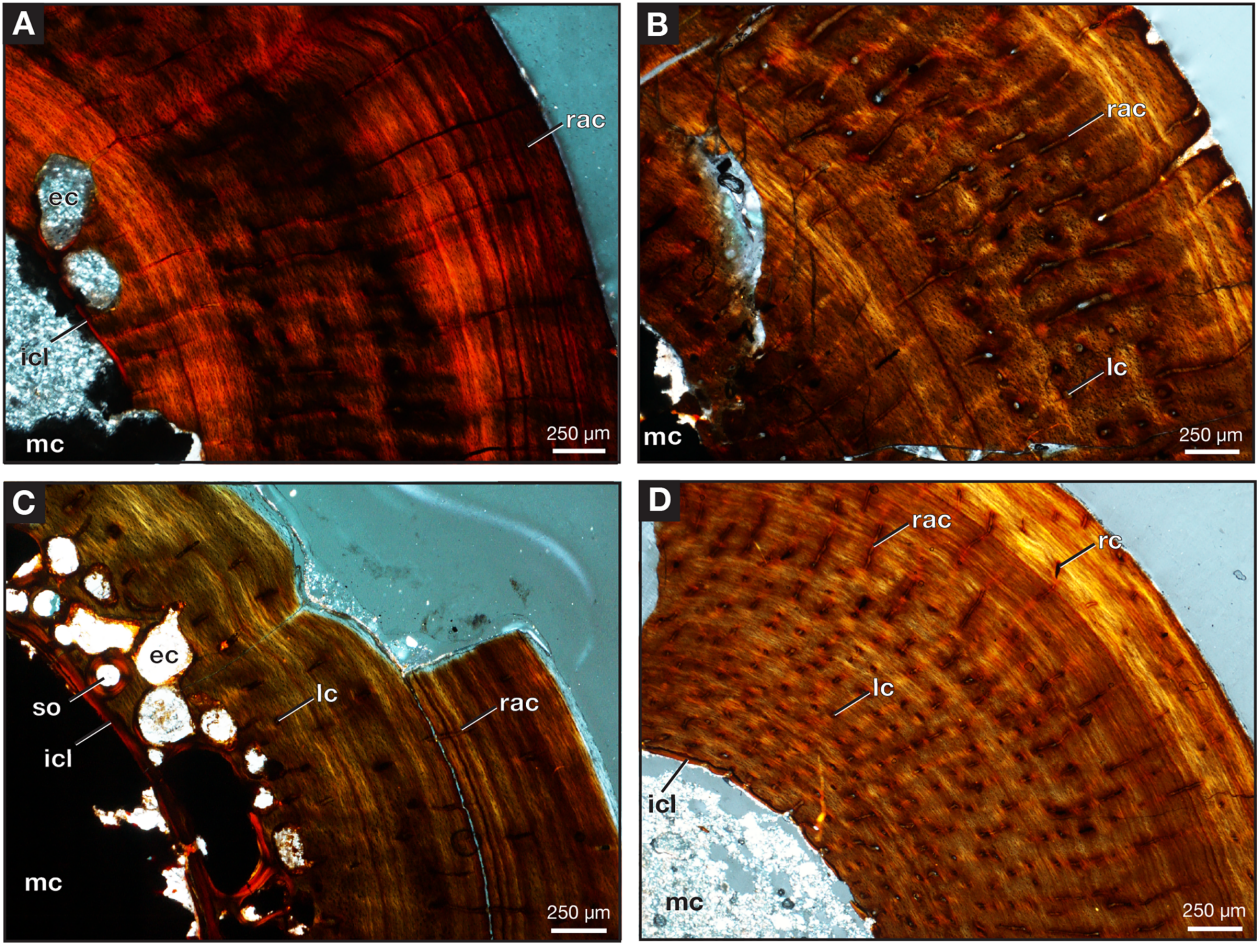
Midshaft of femora under cross-polarized light. Close-up of *Varanops* femora (A) ROMVP 87396, (B) ROMVP 87797, (C) ROMVP 87798. (D) Close-up of *Mesenosaurus* femur, ROMVP 87799. Abbreviations: ec, erosion cavities; icl, inner circumferential lamellae; lc, longitudinal canals; mc, medullary (marrow) cavity; rac, radial canals; rc, reticular canals; so, secondary osteon.

One partial left femur (ROMVP 87396) attributed here to *Varanops* was sectioned near the midshaft and was observed to have lamellar bone near the periphery of the cortex (Fig. 9A), which is identified from the thin layers of lamellae (Francillon-Vieillot et al., 1990; McFarlin, 2006). Huttenlocker & Shelton (2020) examined a few *Varanops* femora from Richards Spur and determined that parallel-fibred tissues were observed near the medullary cavity of the cortex which transition to lamellar tissues, also termed as PF-LAM (McFarlin et al., 2016); thus, our results support previous findings. There are a few radial canals dispersed throughout the cortex (Fig. 10A). Growth marks, specifically lines of arrested growth (LAGs), were found within the cross-section, with approximately 10 lines and two annuli present under normal light. Interestingly, the majority are found near the periphery of the cortex, which is known as the external fundamental system (EFS; Evans et al., 2015) (Fig. 9C). This EFS, a small cross-sectional area with multiple growth lines positioned close together, characterizes the slowing of growth and represents the skeletal maturity of the specimen (Woodward, Horner & Farlow, 2011; Evans et al., 2015). Additionally, large erosion cavities were found in the cortical wall of the medullary cavity. Longitudinal vascular canals were present as circular rows within the center of the cortex. Primary osteons were present within the cortex, while no secondary osteons found (Fig. 9B). The midshaft diameter of the complete femur (ROMVP 87380) is similar to the two femora (OMNH 73758 and UWBM 89463) described by Huttenlocker & Shelton (2020), which were around 10 mm, while the partial femur examined here had a slightly larger diameter, 12 mm. The femora examined by Huttenlocker & Shelton (2020) were found to have five to six growth lines (including LAGs and annuli). We were not able to histologically examine the new articulated specimen (ROMVP 87380) since only the CT data were available; thus, we interpret the similar diameter of the midshaft to indicate that it may have a similar number of LAGs and likely similar in age to the femur of Huttenlocker & Shelton (2020).

Two additional midshafts were sectioned, the first being the proximal half of a left femur (ROMVP 87797) with an approximate shaft diameter of 12 mm (Figs. 9D–9E) and showing the presence of 10 growth lines (five annuli and five LAGs) and no secondary osteons, while the second proximal half of a right femur (ROMVP 87798) with a shaft diameter of 10 mm having 11 growth lines (seven annuli and four LAGs; Figs. 9F–9G) and secondary osteons being present near the medullary cavity, above the inner circumferential lamellae (Fig. 10C). Interestingly, the transverse cross-sections near the pocketing and ridges representing the area of the 4^th^ trochanter, revealed cancellous (spongy) bone with no growth lines present within the outer cortex shows the presence of trabecular structure rather than lamellar bone (Fig. 7C). It is therefore difficult to tell if this area was remodeled as the ridges became more prominent ontogenetically or if the internal trabecular structure prevented the formation of the LAGs.

For comparison, we sectioned the proximal half of a left femur of *Mesenosaurus* (ROMVP 87799). The femur of this taxon, like the known femur of its close relative *Mycterosaurus longipes* (Berman & Reisz, 1982, Fig. 9A) is readily distinguishable from that of *Varanops* by having a relatively indistinctive fourth trochanter and no transverse ridges below the intertrochanteric fossa (Figs. 7D–7E). The thin section revealed the presence of cortical bone throughout the periphery of the bone with a bit of spongy bone in the area of the internal trochanter and none in the area of the fourth trochanter. The midshaft diameter of 7 mm revealed 17 growth lines, including six annuli and eight LAGs, with the external fundamental system present in the periphery of the cortex (Figs. 9H–9I). Additionally, the *Mesenosaurus* femur appears to have a thicker cortex and higher organization of tissues with radiating longitudinal vascular canals (Fig. 10D), compared to the *Varanops* femora, which is similar to the findings of Huttenlocker & Shelton (2020).

## Discussion

Here we have provided important additional information on varanopid skeletal remains recovered from Dolese Brother Limestone Quarry near Richards Spur, which can be assigned with confidence to *Varanops brevirostris*. In addition to the revised diagnosis by Campione & Reisz (2010) which focused mainly on cranial elements, the morphology of the blade-like neural spines, the distinctive v-shaped gap between the last presacral and first sacral spines, the concave posterior surface of the femur’s distal end, and the deeply excavated pubis within the pelvic girdle are all features that are most likely diagnostic for *Varanops brevirostris* (Williston, 1911).

Various members of Varanopidae have historically switched back and forth between synapsids and diapsids reptiles (Reisz & Modesto, 2007), some authors have even claimed archosaurian relationships for *Mesensaurus* and *Varanodon*, respectively (Ivachnenko & Kurzanov, 1979; Reig, 1967). A recent phylogenetic analysis has suggested again that varanopids may be diapsid reptiles (Ford & Benson, 2020). Although interesting, the latest iteration of varanopids as diapsids requires additional investigation, and we therefore prefer to consider them as synapsids. Interestingly, among early amniotes, only synapsids appear to have evolved elongated neural spines, as seen here (Mann & Reisz, 2020).

The new materials are of sufficient quality and detail to confirm that this taxon is present at the Richard Spur locality and is indistinguishable from those from the genotype from the famous *Cacops* bonebed from the early Permian of Texas. It is clear from the femur of ROMVP 87380 that this new material fits within the known size range shown of *V. brevirostris* (Romer & Price, 1940, pg. 464, Table 4), where the smallest femur was 87 mm in length while the largest being slightly over 100 mm in length. The ventral side of the femur exhibits transverse pocketing and ridges below the intertrochanteric fossa, similar to the *Varanops* identified from Mud Hill (Campione & Reisz, 2010), which are located in the usual position of the 4^th^ trochanter, generally interpreted to be the area of insertion of locomotory muscles (caudofemoralis muscle). The external surface in combination with the spongy bone below may be related to the locomotory strategies of this taxon, possibly to resist different types of tension, transfer the load imposed by caudofemoralis muscles and ultimately strengthen the bone (Currey, 2002; Kivell, 2016). Ontogenetically there is an increase in the robustness and size of the ridges and grooves within this area. The smaller and more juvenile *Varanops* femur had a less well-developed transverse ridge, which was only present below the internal trochanter and did not traverse the ventral surface of the bone. This suggests that this area may have changed in morphology as the individual became bigger and older, with more trabecular bone forming deeper within the medullary cavity. Unfortunately, the femora of other varanodontines are either not preserved or poorly known, and we are unable to determine at this time if this character is only present in *Varanops* or is more broadly distributed among members of this clade of varanopids. However, it is apparent that this morphology is distinct from the mycterosaurine varanopids, such as *Mesenosaurus*, which has a poorly formed fourth trochanter.

It is currently not possible to discern this unique, fine morphology of the transverse ridges in specimens from the *Cacops* bonebed (Williston, 1911) because the calcite rich sediment that encrusted the bones at this locality were difficult to separate from the bone surface using the method of preparation during the early 20^th^ century. Unfortunately, all of the currently available specimens have been prepared mechanically at that time, resulting in these external features not being well-preserved. Nevertheless, the presence of these types of transverse ridges and pits appears to be an interesting feature of *Varanops* since it is now known to be present in both the Richards Spur and Mud Hill specimens (Campione & Reisz, 2010).

Additionally, the specimens found at Richards Spur, including the femur and tibia are practically indistinguishable from the *V*. *brevirostris* specimens from the *Cacops* bonebed locality in Baylor County, Texas (Williston, 1911), but the latter limb bones are more gracile than the *Varanops* material from Mud Hill (Campione & Reisz, 2010). Based on previous estimations, we suggest that this new material belonged to an individual that was up to 1.5 m in length and about 25 kg in mass (Romer & Price, 1940). Additionally, the femur may have between 6 to 10 growth lines (including LAGs and annuli), since it has a similar diameter to the two femora (OMNH 73758 and UWBM 89463) examined by Huttenlocker & Shelton (2020) which were also from Richards Spur, while the partial femur histologically sampled in this study was larger in diameter with a greater number of LAGs, and the presence of the external fundamental system at the outer edge of the cortex which represents a slowdown in skeletal growth (Cullen et al., 2021; Woodward, Horner & Farlow, 2011).

When compared to the known terrestrial vertebrate assemblages of the early Permian, we see a distinct difference in their compositions from what we have at Richards Spur. Despite the exceptional diversity and abundance of fossil materials, the top predators in this upland community are significantly smaller than those commonly found in the lowland deltaic and floodplain localities of Texas and Oklahoma. Overall, we have an interesting assemblage of small to medium-sized apex predators preserved at Richards Spur, two temnospondyl amphibians with relatively large heads and short bodies, and two larger amniotes with relatively more gracile bodies. This size difference in the apex predators of Richards Spur is unlikely to be the result of taphonomic effects because we have the preservation of fragmentary skeletal elements or even fragments of skeletal elements that can be readily identified. They all point to a significant difference between the faunal assemblage at Richards Spur and all other localities in the early Permian. This includes the unusual fossil assemblage at the *Cacops* bonebed.

The faunal assemblage of Richards Spur has limited similarities to the *Cacops* bonebed (Williston, 1910), with the famous armored dissorophid *Cacops* being restricted to these two localities (MacDougall et al., 2017). In addition to the presence of *Varanops*, two small, but taxonomically distinct caseids have also been found at these localities, and the captorhinid, cf. *Captorhinus aguti* was also present at the *Cacops* bonebed. However, the interesting difference between these two famous localities is the relative abundance of varanopids and reptiles. While the dissorophid *Cacops* is now known to be relatively abundant at both localities, *Varanops*, while abundant at the *Cacops* bonebed, remains a less common component of the Richards Spur assemblage. Instead, the Richard Spur locality has recently yielded numerous well-preserved skulls and postcranial elements of another varanopid, the smaller more gracile predator *Mesenosaurus efremovi*. Interestingly, the most common reptile at Richards Spur is *Captorhinus aguti*, with other captorhinids being less abundant, while reptilian remains are particularly rare at the *Cacops* bonebed. It is unfortunate that this famous bonebed from the early Permian of Texas is no longer available for studies of its depositional environment and taphonomy since it has become inaccessible under Lake Kemp as a result of damming by the Waggoner ranch (Anderson, Scott & Reisz, 2020).

## Conclusions

This study provides additional information, including a new articulated pelvic region and various isolated vertebrae and limb bones, of the large varanopid *Varanops brevirostris*, recovered from the Dolese Brother Limestone Quarry near Richards Spur. Available evidence indicates that it is indistinguishable from the genotype from the *Cacops* bonebed. The locality has been previously found to be populated mainly by two large dissorophoid predators, *Cacops* and *Acheloma*, and fragmentary remains of the large sphenacodontid *Dimetrodon* have been reported to also be present. Together with previous materials tentatively assigned to *Varanops*, there are now multiple specimens (a minimum of seven individuals) of this taxon at the Richards Spur locality, including the five from the current study. Although appearing to be still somewhat less abundant than *Cacops* and *Acheloma*, this taxon does not appear to be as rare as previously thought. Incorporating neutron computed tomography of the articulated skeleton with histological analyses of isolated femora, we were able to show that the articulated specimen may have belonged to an adult individual with the presence of multiple annuli and lines of arrested growth, as well as the presence of an external fundamental system in the periphery of the cortex of the femora.

## Institutional Abbreviations

FMNH: (WM, UR, UC) Field Museum of Natural History, Chicago, IL
OMNH: Sam Noble Oklahoma Museum of Natural History, University of Oklahoma, Norman, OK, USA
ROMVP: Royal Ontario Museum, Toronto, ON
UWBM: University of Washington Burke Museum of Natural History and Culture, Seattle

## Additional Information and Declarations

### Data Availability

The following information was supplied regarding data availability: Unprocessed 16-bit TIFF slices of ROMVP 87380 are available at MorphoBank: http://dx.doi.org/10.7934/P4455.
